# Developmental and reproductive toxicity of a recombinant protein subunit COVID-19 vaccine (ZF2001) in rats

**DOI:** 10.1038/s41541-023-00673-3

**Published:** 2023-05-24

**Authors:** Yisheng Song, Jinjin Shao, Guangbiao She, Wanqiang Lv, Guoyu Chen, Jing Liu, Lili Zhang, Chengda Zhang, Jiahong Wang, Ruiyu Tian, Lianpan Dai, George F. Gao, Enqi Huang, Lijiang Zhang

**Affiliations:** 1 Center of Safety Evaluation and Research, Hangzhou Medical College, Hangzhou, 310053 China; 2 Key Laboratory of Drug Safety Evaluation and Research of Zhejiang Province, Hangzhou Medical College (Zhejiang Academy of Medical Sciences), Hangzhou, 310053 China; 3 Engineering Research Center of Novel Vaccine of Zhejiang Province, Hangzhou Medical College, Hangzhou, 310000 China; 4 Anhui Zhifei Longcom Biopharmaceutical Co., Ltd, Hefei, 230088 China; 5 CAS Key Laboratory of Pathogen Microbiology and Immunology, Institute of Microbiology, Chinese Academy of Sciences, 100101 Beijing, China

**Keywords:** Drug safety, Drug development

## Abstract

ZF2001, a protein subunit vaccine against coronavirus disease 2019 (COVID-19), contains recombinant tandem repeat of dimeric receptor-binding domain (RBD) protein of the SARS-CoV-2 spike protein with an aluminium-based adjuvant. During the development of this vaccine, two nonclinical studies were conducted to evaluate female fertility, embryo-fetal development, and postnatal developmental toxicity in Sprague‒Dawley rats according to the ICH S5 (R3) guideline. In Study 1 (embryo-fetal developmental toxicity, EFD), 144 virgin female rats were randomly assigned into four groups and received three doses of vaccine (25 μg or 50 μg RBD protein/dose, containing the aluminium-based adjuvant), the aluminium-based adjuvant or a sodium chloride injection administered intramuscularly on days 21 and 7 prior to mating and on gestation day (GD) 6. In Study 2 (pre- and postnatal developmental toxicity, PPND), ZF2001 at a dose of 25 μg RBD protein/dose or sodium chloride injection was administered intramuscularly to female rats (*n* = 28 per group) 7 days prior to mating and on GD 6, GD 20 and postnatal day (PND) 10. There were no obvious adverse effects in dams, except for local injection site reactions related to the aluminium-based adjuvant (yellow nodular deposits in the interstitial muscle fibres). There were also no effects of ZF2001 on the mating performance, fertility or reproductive performance of parental females, embryo-fetal development, postnatal survival, growth, physical development, reflex ontogeny, behavioural and neurofunctional development, or reproductive performance of the offspring. The strong immune responses associated with binding and neutralising antibodies were both confirmed in dams and fetuses or offspring in these two studies. These results would support clinical trials or the use of ZF2001 in maternal immunisation campaigns, including those involving women with childbearing potential, regardless of pregnancy status.

## Introduction

The coronavirus disease 2019 (COVID-19) pandemic caused by severe acute respiratory syndrome coronavirus 2 (SARS-CoV-2) is still ongoing worldwide. Globally, as of December 2022, more than 646 million people have been diagnosed with COVID-19, with ~6.6 million deaths, as reported by the World Health Organization (WHO)^1^. Safe and effective vaccines are critical to protecting susceptible populations against COVID-19 and ending the pandemic. Therefore, several COVID-19 vaccines have been developed and approved for use at an accelerated pace in many countries, including mRNA vaccines, adenovirus vector vaccines, inactivated vaccines and protein subunit vaccines^2–6^. All these vaccines have played a considerable role in preventing infection and reducing morbidity related to SARS-CoV-2.

Although the total number of vaccination doses globally to date has exceeded 13.0 billion, the public remains concerned and hesitant towards vaccination, especially pregnant and lactating women, who are usually excluded from the clinical trials of the COVID-19 vaccines^7,8^. Theoretically speaking, pregnant and lactating women may be more likely to experience an increased risk of severe COVID-19 infection due to the alterations in adaptive immunity and mechanical and physiological characteristics associated with pregnancy^9,10^. Specifically, when compared with their non-infected pregnant counterparts, pregnant patients are threefold more likely than their infected nonpregnant counterparts to be admitted to intensive care units (ICUs) and receive ventilator support or extracorporeal membrane oxygenation (ECMO), and their mortality rate is higher in addition to the risk of obstetric complications, such as preterm birth in particular^11–13^. Given the increased risk associated with COVID-19 during pregnancy, many pregnant women have decided to accept the vaccine even though critical data on the benefits and risks in pregnant individuals have been lacking, making it imperative to include pregnant or lactating women in COVID-19 vaccine trials^14^.

ZF2001 is a protein subunit vaccine that has been approved for emergency use in China, Uzbekistan, Indonesia, and Columbia, and more than 200 million doses in humans have been administered. Unlike other vaccine candidates studied in clinical trials that aim mainly at the whole virus or spike protein^15^, ZF2001 targets the receptor-binding domain (RBD) of the SARS-CoV-2 S protein^6,16^. The RBD is responsible for engagement with its cellular receptor, angiotensin-converting enzyme 2 (ACE2), and it is an attractive vaccine target to induce immune responses by blocking receptor binding^16,17^. ZF2001 is generated by an RBD-dimer protein produced in Chinese hamster ovary (CHO) cells adjuvanted with aluminium hydroxide (Al (OH)_3_)^18^. The resulting vaccine ZF2001 demonstrated safety and immunogenicity in adults in phase 1 (NCT04445194, NCT04550351) and phase 2 (NCT04466085) clinical trials and showed a clinical efficacy of 81.4% in adults in a multinational phase 3 clinical trial (NCT04646590)^6,19^. However, pregnant and lactating women were excluded from the initial vaccine clinical trials. The related guidelines of the WHO, the International Council for Harmonization of Technical Requirements for Pharmaceuticals for Human Use (ICH), and the National Medical Products Administration of China (NMPA) all describe expectations for the nonclinical developmental and reproductive toxicity (DART) study that are necessary before performing a clinical trial of a vaccine in pregnant women^20,21^. Here, we reported two nonclinical DART studies evaluating the effects on female fertility, embryo-fetal development, and prenatal and postnatal developmental toxicity associated with ZF2001 in Sprague‒Dawley rats. These data support the initiation of a clinical trial or the use of ZF2001 in pregnant and lactating women.

## Results

### General toxicity of F0 female rats

**Fig. 1:**
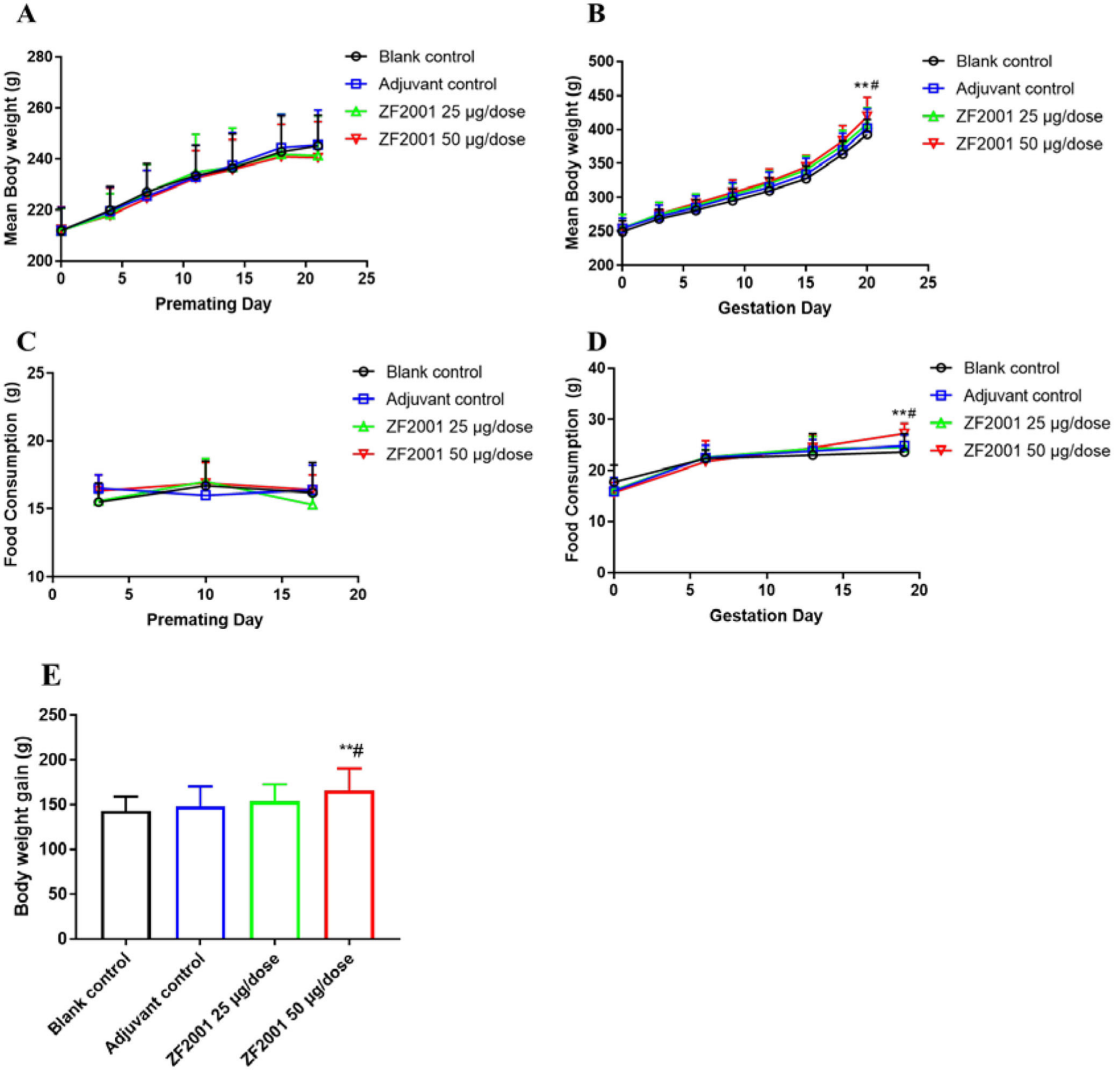
Mean body weight and food consumption of F0 females in Study 1. Data are expressed as the mean ± SD, and were analysed using one-way ANOVA. Compared with blank control group, **one-way ANOVA *p* < 0.01; compared with adjuvant control group, ^#^one-way ANOVA *p* < 0.05. **A** Body weight of female rats during the premating days; **B** Maternal body weight during gestation days in rats; **C** Food consumption of female rats during the premating days; **D** Food consumption during gestation days in maternal rats. **E** Body weight gain from GD 0 to 20 in maternal rats.

**Fig. 2:**
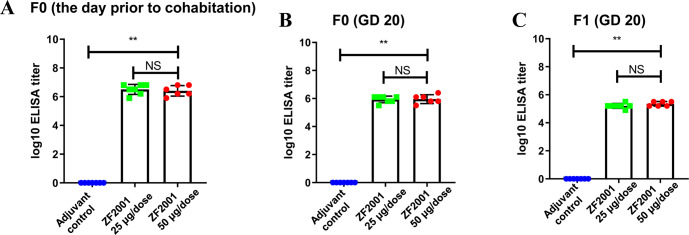
The titres of anti-NPC-RBD-binding antibody in Study 1. Compared with the adjuvant control group, **one-way ANOVA *p* < 0.01. **A** The titres of anti-NPC-RBD-binding antibody of F0 females on the day prior to cohabitation; **B** The titres of anti-NPC-RBD-binding antibody of F0 females on GD 20; **C** The titres of anti-NPC-RBD-binding antibody of F1 pups on GD 20.

In Study 1 and Study 2, all F0 female rats in each group subjected to a dose of 25 μg and/or 50 μg/dose of the ZF2001 vaccine were noted to exhibit khaki-yellow nodular deposits in the interstitial muscle fibres at the administration site, which was considered a typical change associated with the aluminium-containing adjuvant in the local administration area based on previous data from our laboratory, rather than the interaction with the antigen of vaccine, and no other general toxicity was observed. The ZF2001 vaccine had no toxic effects on the body weight and food intake of F0 female rats during the whole period of both studies (Fig. 1 and Supplementary Fig. 1). During the gestation of F0 females in Study 1, body weights (on GD 20, the percentage change relative to the adjuvant and blank control group was 4.3% and 6.8%, respectively), body weight gain from GD 0 to 20 (up to 11.8% and 15.7% compared with the adjuvant and blank control group, respectively) and food consumption (on GD 19; the percentage change relative to the adjuvant and blank control group was 9.7% and 15.3%, respectively) in the ZF2001 50 μg/dose group were significantly higher than those in the adjuvant (up to 4.3% control) and blank control group (up to 6.8% control) (Figs. 1B, D and 2E) and were not considered to be adverse effects. Similar nonadverse increases in body weight and food intake at selected time points were also observed in the ZF2001 vaccine group in Study 2 (Supplementary Fig. 1C, D). There were no vaccine-related changes observed in the results of the gross examination of maternal thoracic and abdominal viscera in either study.

### Female fertility and embryo–fetal development toxicity in rats

In Study 1 (EFD), female fertility data, including the mating index, pregnancy rate, and fertility index, were unaffected by maternal treatment with the ZF2001 vaccine (Table 1).

**Table 1:** Summary of fertility and caesarean section data in main study cohort of Study 1 (EFD).

Indicators	Blank control	Adjuvant control	ZF2001 25 μg/dose	ZF2001 50 μg/dose
Female mating index	17/24 (70.8%)	21/24 (87.5%)	19/24 (79.2%)	19/24 (79.2%)
Pregnancy rate	16/17 (94.1%)	21/21 (100%)	17/19 (89.5%)	17/19 (89.5%)
Fertility index	16/24 (66.7%)	21/24 (87.5%)	17/24 (70.8%)	17/24 (70.8%)
Weight of uterus and embryo (g)	77.75 ± 14.20	80.27 ± 17.03	78.75 ± 21.97	84.39 ± 15.90
Placenta weight (g)	0.49 ± 0.05	0.51 ± 0.07	0.55 ± 0.07*	0.55 ± 0.08*
Corpus lutea	18.25 ± 3.49	17.62 ± 3.17	16.18 ± 2.43	16.88 ± 2.55
Implantation sites	13.88 ± 2.33	13.90 ± 3.14	14.06 ± 2.75	14.76 ± 1.71
Live fetuses	13.44 ± 2.39	13.71 ± 3.24	13.12 ± 3.87	14.00 ± 2.85
Live fetuses rate (%)	96.8 ± 4.7	98.5 ± 3.3	91.4 ± 17.9	94.8 ± 15.9
Dead fetuses	0.00 ± 0.00	0.05 ± 0.22	0.12 ± 0.33	0.06 ± 0.24
Dead fetuses rate (%)	0.0 ± 0.0	0.4 ± 1.8	0.9 ± 2.6	0.3 ± 1.4
Resorptions	0.44 ± 0.63	0.14 ± 0.36	0.82 ± 1.74	0.71 ± 2.20
Resorptions rate (%)	3.2 ± 4.7	1.1 ± 2.9	7.7 ± 16.4	4.9 ± 15.6
Preimplantation loss rate (%)	21.7 ± 17.4	19.7 ± 18.5	13.1 ± 14.6	11.8 ± 9.4
Postimplantation loss rate (%)	3.2 ± 4.7	1.5 ± 3.3	8.6 ± 17.9	5.2 ± 15.9
Sex ratio (% male)	51.5 ± 8.3	48.9 ± 15.2	49.1 ± 14.3	47.9 ± 13.0
Fetal body weight (g)	3.82 ± 0.21	3.82 ± 0.25	3.68 ± 0.46	3.82 ± 0.36
Fetal body length (cm)	3.77 ± 0.08	3.78 ± 0.11	3.70 ± 0.19	3.75 ± 0.12
Fetal tail length (cm)	1.15 ± 0.03	1.15 ± 0.04	1.14 ± 0.07	1.15 ± 0.04

Data in each group presented as mean per litter standard deviation.

Female mating index = Females mated/Total females × 100%.

Pregnancy rate = Pregnant females/Females mated × 100%.

Fertility index = Pregnant females/Total females × 100%.

Live fetuses rate (%) = Live fetuses/Implantation sites × 100%.

Dead fetuses rate (%) = Dead fetuses/Implantation sites × 100%.

Resorptions rate (%) = Resorptions/Implantation sites × 100%.

Preimplantation loss rate (%) = (Corpus lutea-Implantation sites)/Corpus lutea × 100%.

Postimplantation loss rate (%) = (Implantation sites-Live fetuses)//Corpus lutea × 100%.

Compare with blank control, *one-way ANOVA *p* < 0.05.

Caesarean section data assessed per litter (Table 1), including the mean number of corpora lutea, implantation sites, resorptions, viable fetuses, weight of uterus and embryo, pre- and postimplantation loss, sex ratio, body weight, body length and tail length of fetuses, showed no adverse effects of treatment with either dose level of the ZF2001 vaccine. The placental weights in the 25 μg and 50 μg/dose groups were higher than that in the control group (one-way ANOVA *p* < 0.05), but there was no significant difference from the adjuvant control (one-way ANOVA *p* > 0.05), and increased placental weight was not considered an adverse change.

After caesarean section, fetuses were evaluated for the potential effects of ZF2001 on fetal morphological development. There were no adjuvant- or ZF2001-related fetal external, visceral, or skeletal malformations, and the incidence of all fetal abnormalities, including malformations, variations and uncategorised abnormalities, in the adjuvant control or ZF2001 vaccine groups was not significantly different from that in the blank control group (Table 2). One case of spontaneous malformation was found both in the blank control and the 50 µg/dose group, which were similar to normal background findings in rats and only occurred in the single fetus and thus were not considered to be related to the ZF2001 vaccine.

**Table 2:** Summary of fetal examination data in EFD study.

Indicators	Blank control	Adjuvant control	ZF2001 25 μg/dose	ZF2001 50 μg/dose
External examination				
Fetuses/litters examined (*n*)	215/16	288/21	223/17	238/17
Total malformations	1/1	0/0	0/0	1/1
Total variations or uncategorised abnormalities	1/1	1/1	0/0	1/1
Mandible, small [M]	1/1	0/0	0/0	0/0
Exencephaly [M]	0/0	0/0	0/0	1/1
Tongue, protruding [V]	1/1	1/1	0/0	1/1
Soft tissue examinations				
Fetuses/litters examined (*n*)	102/16	138/21	107/17	116/17
Total malformations	0/0	0/0	0/0	1/1
Total variations or uncategorised abnormalities	0/0	0/0	0/0	0/0
Brain, misshapen [M]	0/0	0/0	0/0	1/1
Skeletal examination				
Fetuses/litters examined (*n*)	113/16	150/21	116/17	122/17
Total malformations	0/0	0/0	0/0	0/0
Total variations or uncategorised abnormalities	88/16	117/21	114/17	107/17
Frontal, incomplete ossification [V]	1/1	0/0	0/0	0/0
Parietal, incomplete ossification [V]	15/10	5/2	4/3	14/8
Interparietal, incomplete ossification [V]	53/14	66/19	89/17	58/17
Occipital, incomplete ossification [V]	6/4	0/0	3/2	0/0
Mandible, incomplete ossification [V]	1/1	0/0	0/0	0/0
Mandible, small [V]	1/1	0/0	0/0	0/0
Metacarpal, incomplete ossification [V]	0/0	1/1	2/1	0/0
Metatarsal, incomplete ossification [V]	2/2	0/0	2/1	0/0
Sternum, incomplete ossification [V]	29/14	34/15	34/14	26/12
Sternum, unossified [U]	61/16	81/18	57/17	72/17
Thoracic vertebral centrum, dumbbell ossification [V]	9/8	19/10	32/14	17/8
Thoracic vertebral centrum, unilateral ossification [U]	1/1	0/0	1/1	0/0
Thoracic vertebral centrum, two-site ossification [V]	4/4	2/2	4/3	8/7
Lumbar vertebra centrum, unossified [U]	1/1	0/0	0/0	0/0
Lumbar vertebra centrum, incomplete ossification [V]	0/0	0/0	0/0	1/1
Sacral vertebra centrum, incomplete ossification [V]	1/1	0/0	2/2	0/0

Data are presented as number of fetuses affected/number of litters affected.

Litters with malformations rate (%) = Litters with malformations/litters examined × 100%.

Litters with variations rate (%) = Litters with variations/litters examined × 100%.

*[M]* malformation, *[V]* variation, *[U]* uncategorised abnormality, – not to observe.

The above results suggested that there is no female fertility or embryo-fetal toxicity effect in rats vaccinated with the ZF001 vaccine before pregnancy.

### Pre-and postnatal development toxicity in rats

In Study 2 (PPND), there were also no effects on the mating index and pregnancy rate of female rats after administration of the ZF2001 vaccine (Table 3), which was similar to the fertility data in Study 1. Additionally, as shown in Table 3, none of the data associated with maternal delivery in the ZF2001 vaccine group, such as parturition rate, gestation length, pups per litter and live pups per litter at birth, showed significant differences from those in the blank control group. Regarding the postnatal pups, there were no adverse effects on the survival rates, body weights throughout lactation, sex ratio, or incidence of external malformation in the ZF2001 group.

**Table 3:** Summary of maternal delivery and pup data in PPND study.

Indicators	Blank control	ZF2001 25 μg/dose
Total females	28	28
Female mating index^a^	19/28 (67.9%)	20/28 (71.4%)
Pregnancy rate^b^	16/19 (84.2%)	16/20 (80.0%)
Parturition rate^c^	16/16 (100%)	16/16 (100%)
Females with unsuccessful delivery^d^	0	0
Gestation length (days)	21.8 ± 0.4	21.6 ± 0.5
Pups per litter at birth	12.56 ± 3.27	12.44 ± 3.43
Live pups per litter at birth	12.50 ± 3.18	12.31 ± 3.34
Survival rate at birth^e^	99.5%	99.0%
Survival rate on PND 4^f^	97.0%	98.5%
Survival rate in lactation (on PND21)^g^	100%	100%
External malformation rate of pups born	0%	0%
Sex ratio of pups(♂/♀)	77/123	87/110
Pup body weight on PND 0 (g)	6.59 ± 0.58	6.89 ± 0.86
Pup body weight on PND 4 (g)	10.73 ± 1.15	10.96 ± 1.24
Pup body weight on PND 7 (g)	17.44 ± 0.97	17.83 ± 1.50
Pup body weight on PND 10 (g)	25.10 ± 1.62	25.60 ± 2.13
Pup body weight on PND 14 (g)	35.71 ± 2.30	36.46 ± 2.93
Pup body weight on PND 17 (g)	43.05 ± 2.82	44.36 ± 3.47
Pup body weight on PND 21 (g)	57.97 ± 4.81	59.66 ± 5.20

Data are expressed as the mean per litter ± standard deviation, number or the percentage.

^a^Female mating index = Females mated/Total females × 100%.

^b^Pregnancy rate = Pregnant females/Females mated × 100%.

^c^Parturition rate = Females that have completed parturition/Pregnant females × 100%.

^d^Unsuccessful delivery includes miscarriage, dystocia, premature, late or incomplete delivery.

^e^Survival rate at birth = Number of pups born alive/Number of pups born × 100%.

^f^Survival rate on PND 4 = Number of live pups on PND 4/Number of pups born alive × 100%.

^g^Survival rate in lactation = Number of live pups on PND 21/Number of live pups after post-cull on PND 4 × 100%.

There was no evidence of toxic effects related to the ZF2001 vaccine (25 μg/dose) on offspring growth, physical development or neurofunctional or reproductive development from preweaning to puberty. There were no effects on the ages at which physical, reflex and sexual developmental signs were attained (Table 4). The results of autonomous activity achieved by the Top Scan animal behaviour analysis system were also not affected by the ZF2001 vaccine when this group was compared with the control (Table 5). Similarly, all pups in the control and ZF2001 groups exhibited a lack of difference in scores of modified Irwin’s behavioural evaluation (Table 6). Regarding the reproductive performance of exposed F1 offspring, there were no adverse ZF2001 treatment-related effects (Table 7). Caesarean section data from the pregnant F1 generation in the ZF2001 vaccine group were comparable to those in the control group and the parental generation (Table 7). It should be noted that the body weights of F1 females after weaning and during gestation day were significantly higher than those in the control group, and this change was considered nonadverse, while the body weights of the F1 male rats were similar to those in the control group (Supplementary Fig. 1A–D). There were no treatment-related food consumption changes observed in the F1 animals (Supplementary Fig. 1E–G).

**Table 4:** Postnatal day when developmental signs were attained in F1 pups in PPND study.

Indicators	Blank control	ZF2001 25 μg/dose
Total litters	16	16
Physical development		
Auricle separation	3.1 ± 0.7	3.4 ± 0.5
Incisor eruption	11.1 ± 0.7	11.0 ± 0.6
Appearance of fur	11.9 ± 0.5	12.0 ± 0.4
Eyes opening	15.0 ± 0.4	15.2 ± 0.5
Pinna unfolding	16.9 ± 0.6	16.8 ± 0.8
Reflex development		
Plane Correction	4.4 ± 0.9	4.2 ± 1.1
Negative geotaxis	8.6 ± 0.8	8.9 ± 1.2
Auditory Startle	13.7 ± 1.4	13.3 ± 0.9
Aerial righting	15.8 ± 0.9	15.8 ± 0.6
Pupillary reflex	15.0 ± 0.4	15.2 ± 0.5
Sexual development		
Vaginal opening	34.0 ± 1.8	33.9 ± 2.3
Preputial separation	40.6 ± 1.0	40.4 ± 1.0

Data presented as mean age (day at which 100% pups attained landmark) per litter ± standard deviation.

**Table 5:** Summary of autonomous activity of F1 offspring rats in PPND study.

Indicators	Blank control	ZF2001 25 μg/dose
Total fetuses	32	32
The total length of the route (mm)	2299.7 ± 1682.8	2898.8 ± 1576.2
Average speed in the box (mm/s)	7.7 ± 5.6	9.7 ± 5.3
Staying time in the central area (s)	4.4 ± 6.8	6.9 ± 11.1
The length of the route in the central area (mm)	99.6 ± 151.3	163.0 ± 206.4
Grooming (times/5 min)	8.6 ± 3.6	7.6 ± 3.1
Standing (times/5 min)	9.0 ± 9.9	10.5 ± 9.5

Data presented as mean per individual ± standard deviation.

**Table 6:** Modified Irwin’s behavioural assessment of F1 offspring rats in PPND study.

Indicators	Classification	Blank control	ZF2001 25 μg/dose
Total fetuses		32	32
Spontaneous activity			
Body position	Normal:	32/32	32/32
Bizarre behaviour	Not found:	32/32	32/32
Restlessness	Not found:	32/32	32/32
Autonomic			
Piloerection	Not found:	32/32	32/32
Abnormal coat	Not found:	32/32	32/32
Palpebral ptosis/closure	Not found:	32/32	32/32
Respiratory rate	Normal:	32/32	32/32
Lacrimation	Not found:	32/32	32/32
Salivation	Not found:	32/32	32/32
Exophthalmos	Not found:	32/32	32/32
Skin colour	Not found:	32/32	32/32
Motor-affective response			
Vocalisation	Not found:	27/32	28/32
	Stress reaction:	5/32	4/32
Urination	Not found:	31/32	30/32
	Stress reaction:	1/32	2/32
Defecation	Not found:	32/32	32/32
Provoked biting	Not found:	32/32	32/32
Transfer arousal	No reaction:	5/32	12/32
	Minor reaction:	12/32	9/32
	Normal:	15/32	11/32
Spatial locomotion	Normal:	32/32	32/32
Touch escape	No reaction:	29/32	30/32
	Minor reaction:	3/32	2/32
Positional passivity	Not found:	32/32	32/32
CNS excitation			
Tremor	Not found:	32/32	32/32
Twitch	Not found:	32/32	32/32
Convulsion	Not found:	32/32	32/32
Muscle tone			
Body tone	Normal:	32/32	32/32
Grip strength	Normal:	32/32	32/32
Sensory-motor response			
Pinna reflex	Normal:	32/32	32/32
Corneal reflex	Normal:	32/32	32/32
Visual placing reflex	Normal:	32/32	32/32
Startle reflex	Normal:	32/32	32/32
Tail-pinch reflex	Normal:	32/32	32/32
Posture			
Tail elevation	Not found:	32/32	32/32
Equilibrium and gait			
Ataxic gait	Not found:	32/32	32/32
Hypotonic gait	Not found:	32/32	32/32
Total gait incapacity	Not found:	32/32	32/32
Surface righting reflex	Normal:	32/32	32/32
Spatial righting reflex	Normal:	32/32	32/32

**Table 7:** Summary of reproductive performance of F1 offspring rats in PPND study.

Indicators	Blank control	ZF2001 25 μg/dose
Total pairs cohabited	16	16
Mated females	16	15
Pregnant females	15	14
Time to mating (days)	3.3 ± 1.7	3.2 ± 0.9
Mating index (%)	100	93.8
Pregnancy (%)	93.8	93.3
Fertility index (%)	93.8	87.5
Corpus lutea	21.2 ± 6.8	23.6 ± 5.2
Implantation	16.2 ± 1.9	16.3 ± 2.5
Live fetuses	14.8 ± 2.1	14.9 ± 2.2
Live fetuses (%)	91.4 ± 9.8	91.5 ± 7.8
Dead fetuses	0.0 ± 0.0	0.0 ± 0.0
Dead fetuses (%)	0.0 ± 0.0	0.0 ± 0.0
Resorptions	1.4 ± 1.7	1.4 ± 1.4
Resorptions (%)	8.6 ± 9.8	8.5 ± 7.8
Preimplantation loss (%)	19.9 ± 15.1	28.4 ± 15.9
Postimplantation loss (%)	8.6 ± 9.8	8.5 ± 7.8

Data in each group presented as mean per litter ± standard deviation.

Mating index = Females mated/females cohabited × 100%.

Pregnancy (%) = Pregnant females/Females mated × 100%.

Fertility index = Pregnant females/females cohabited × 100%.

Live fetuses (%) = Live fetuses/Implantation sites × 100%.

Dead fetuses (%) = Dead fetuses/Implantation sites × 100%.

Resorptions (%) = Resorptions/Implantation sites × 100%.

Preimplantation loss (%) = (Corpus lutea-Implantation sites)/Corpus lutea × 100%.

Postimplantation loss (%) = (Implantation sites-Live fetuses)//Corpus lutea × 100%.

### Binding and neutralising antibody response

In the EFD study (i.e., Study 1), serological RBD-binding IgG (anti-NCP-RBD-binding antibodies) was detected in all F0 females in the immunogenicity cohort on the day prior to cohabitation and on GD 20 in the ZF2001 groups, with a geometric mean titre (GMT) of 10^6^ (Fig. 2A, B). Thus, anti-NCP-RBD-binding antibodies were passively transferred through the umbilical cord during pregnancy from the F0 females to the F1 pups, according to the high GMT of anti-NCP-RBD-binding antibodies in F1 pups on GD 20 (Fig. 2C). There was no significant difference in the GMT of anti-NCP-RBD-binding antibodies between the two groups subjected to ZF2001 treatment with 25 or 50 μg/dose. As expected, anti-NCP-RBD-binding antibody was not detectable in the blood samples of F0 females or in their fetuses or in the sodium chloride control and adjuvant control groups.

**Fig. 3:**
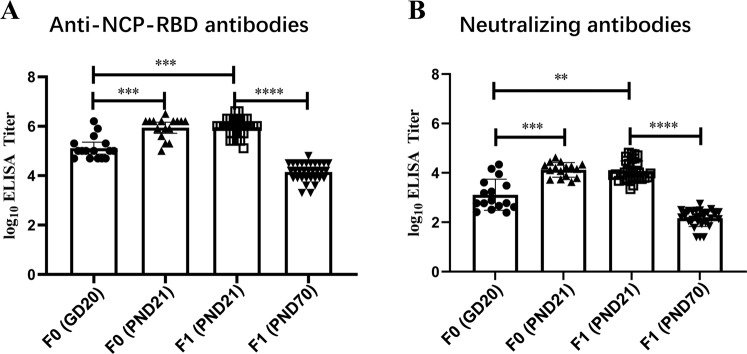
The titres of anti-NPC-RBD-binding and neutralising antibodies of the ZF2001 group in Study 2. Compared with each other, **one-way ANOVA *p* < 0.01, ***one-way ANOVA *p* < 0.001, ****one-way ANOVA *p* < 0.0001. **A** The titres of anti-NPC-RBD-binding antibodies of F0 females on GD 20, PND21, and F1 pups on PND21, PND70; **B** The titres of neutralising antibodies of F0 females on GD 20, PND21, and F1 pups on PND21, PND70.

In the PPND study (i.e., Study 2), serological RBD-binding IgG was detected in all F0 mated or pregnant females on GD 20 and PND 21 and in F1 pups on PND 21 and PND 70 in the ZF2001 group with similar GMTs as those in the EFD study (Fig. 3A). In addition, administration of ZF2001 elicited SARS-CoV-2 neutralising antibody responses in F0 females and all their offspring. Neutralising antibody titres against SARS-CoV-2 were detected in F0 females on GD20, which was 14 days following the second dose administration, and titres of neutralising antibody remained elevated on PND 21, which was 11 days following the fourth dose administration. Similar to the dams, high titres of SARS-CoV-2 neutralising antibody were observed in all offspring (F1 pups on PND 21 and PND 70) in the ZF2001 group (Fig. 3B), and anti-NCP-RBD antibody and SARS-CoV-2 neutralising antibody were not detectable in the blood samples of dams and their pups in the sodium chloride control group or the adjuvant control group.

## Discussion

Animal experimental research is currently one of the best tools for assessing the developmental and reproductive toxicity of vaccines in humans and provides credible data to support clinical studies in pregnant women and reproductive-aged women^22,23^. These two rat DART studies, EFD and PPND, of the ZF2001 vaccine were conducted following recently updated ICH S5 (R3) guideline on nonclinical safety evaluation of vaccines for infectious disease^21^ and related requirements in the 2006 FDA Guidance^24^, and NMPA guidelines on preclinical safety evaluation of prophylactic biological products. The rat model used in this study is widely accepted and the most often used rodent species for DART testing, with significant available historical background data on the entire reproductive spectrum^25^. Thus, the nonclinical DART study results described in this manuscript provide important and critical data on ZF2001 in pregnant and lactating rats and associated effects with potential human risk.

In the EFD study, the administration of 1- or 2-fold the human dose (i.e., 25 µg/dose), which was greater than 50 or 100-fold relative to body weight and determined after body surface area conversion, given to female rats that were subjected to ZF2001 treatment prior to pairing and during gestation did not affect female mating performance or fertility. Litter data were unaffected by maternal treatment, as assessed by the numbers of corpora lutea, implantations, resorptions, live fetuses, sex ratio, placental weight, fetal weight and length, which were similar among. Furthermore, detailed fetal examination did not reveal any major or minor external, soft tissue, and skeletal abnormalities or variations considered to be related to treatment.

In the PPND study, gestation and parturition were unaffected by treatment, with all pregnant females producing live litters of similar sizes, offspring survival rates and sex ratios. No treatment-related macroscopic necropsy findings were observed in females or their offspring. In addition, there was no obvious toxicity to offspring development, such as that associated with appearance development, body weight, physiological and reflex development, behavioural development, sexual development or fertility, in F1 generation rats. Similar to our two DART studies, it was reported that a lack of nonclinical reproductive and developmental toxicity was revealed to be associated with the mRNA-based COVID-19 vaccine^23^.

In these two DART studies, F0 generation female rats inoculated with the ZF2001 vaccine showed the production of high titres (GMT: 10^5^–10^6^; serum antibody-positive rate was 100%) of binding antibody IgG (anti-NCP-RBD) and/or pseudovirus neutralising antibody during pregnancy or lactation. In addition, the resulting antibodies were demonstrated to have transferred into F1 rats through placental transport and lactation. Our antibody results from the rat studies support that pregnant women or nursing mothers who received the ZF2001 vaccine could give their offspring protection against the SARS-CoV-2 virus through vertical transmission.

Researchers have shown that pregnant women are at increased risk of severe COVID-19, and the development and administration of COVID-19 vaccines could help mitigate this risk^26,27^. Data have demonstrated that the maternal transfer of COVID-19 vaccine-induced antibodies to neonates, which are measured in umbilical cord blood, may offer protection to infants^28^. However, during early years, clinical trials were not often conducted in pregnant women, and thus, critical data describing the benefit/risk in pregnant individuals have been lacking. Moreover, there have been many studies demonstrating the safety and immunogenicity of influenza vaccination in pregnant women, strengthening evidence of maternal antibody transfer and confirming the clinical benefit of maternal influenza vaccination both for the mother and the infant over any perceived vaccination risks^29,30^.To date, tens of thousands of pregnant people have received COVID-19 vaccines globally, including in the U.S., Canada, the U.K., and Israel^7^. Consistent with our results from clinical trials^6,19^, these vaccines offer a favourable level of severe COVID-19 protection, and there have been no suggestive reports of any safety concerns. Data available in pregnant women indicated that some COVID-19 vaccines, such as mRNA COVID-19 vaccines, that is, those from Pfizer/BioNTech (Mainz, Germany) or Moderna (Cambridge, MA), have been well tolerated and could lead to high levels of antibodies passed to infants^31–33^.

Since its emergency use was approved in March 2021, the cumulative dose of ZF2001 has exceeded 350 million doses in China, Uzbekistan, Indonesia and Colombia. However, the absence of specific clinical trial data on the use of ZF2001 in pregnant women resulted in limited use of the ZF2001 vaccination by pregnant women, and this vaccine hesitancy is putting mothers and fetuses at higher risk of pregnancy complications related to COVID-19. Our nonclinical findings presented in these two DART studies completed in rats, coupled with the positive profile of efficacy and safety in nonpregnant women in clinical trials of ZF2001^6,19^, strengthen the confidence in the safety of ZF2001 and support its clinical use in pregnant and lactating women. Moreover, these data from DART studies have already supported the approval of ZF2001 for marketing in China and Uzbekistan. However, considering the uncertainty of the extrapolation of results from animals to humans due to the difference in species, more clinical data are needed to verify the safety of this vaccine in pregnant women and maternal populations. Based on the fact that the COVID-19 epidemic is now under stable control globally, clinical safety information may be expected primarily from real-world data of pregnant or maternal women who have used ZF2001. The safety of this vaccine for vaccination in women of childbearing age, pregnancy, and lactation should be further clarified after a full integration of nonclinical and clinical safety data was evaluated.

## Methods

### Animals

The study was conducted by the Center of Safety Evaluation and Research, Hangzhou Medical College, Hangzhou, Zhejiang Province, China. Sprague‒Dawley rats were group housed (up to 4 per cage) in single-sex groups until paired for mating, at which time females were housed 1:1 with a nontreated breeding male. The female rats were individually housed through gestation and lactation following evidence of mating. Rats were provided with a complete rodent breeding diet and locally sourced water (softened and filtered) ad libitum. Environmental conditions throughout the studies were set to maintain a relative humidity of 43–56% and temperature of 21.5–23.0 °C along with the room lighting set to provide a 12 h light/dark cycle.

The facility where these studies were conducted is accredited by the Association for Assessment and Accreditation of Laboratory Animal Care International (AAALAC, #001489). All animal care and experimental procedures were conducted in compliance with guidelines for the care and use of laboratory animals and the relevant regulations of the Institutional Animal Care and Use Committee (IACUC) and approved by the IACUC (approval number: GLP-2020-105/1 and GLP-2021-076).

### Vaccine

The vaccine was jointly developed by the Institute of Microbiology, the Chinese Academy of Sciences, and Anhui Zhifei Longcom Biopharmaceutical. The vaccine was manufactured according to good manufacturing practice (GMP) guidelines by Anhui Zhifei Longcom Biopharmaceutical. The recombined vaccine encoded the SARS-CoV-2 RBD antigen (residues 319-537, accession number YP_009724390), with two copies in tandem repeat dimeric form, and it was manufactured in the CHOZN CHO K1-cell line (Sigma-Aldrich Trading, China) as a liquid formulation containing 25 or 50 μg per 0.5 ml in a vial, with aluminium hydroxide (0.5 mg/ml) in sodium chloride injection as the adjuvant. The blank control consisted of sodium chloride injection (Zhejiang Guojing Pharmaceutical, China), and the adjuvant control contained only aluminium hydroxide in sodium chloride injection. Vaccines and adjuvants were stored at 2–8 °C before use.

### Study design

Study designs were developed mainly in accordance with ICH guidelines S5 (R3)^21^.

**Fig. 4:**
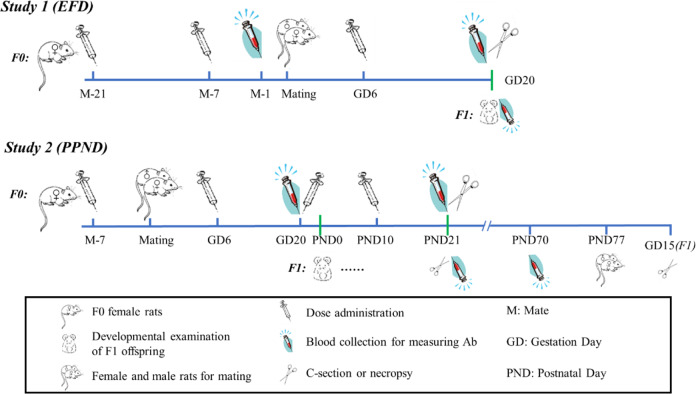
Overview of study design for Study 1 and Study 2. Virgin female rats were administered three or four intramuscular doses of sodium chloride injection (Blank control), aluminium hydroxide adjuvant (Adjuvant control) or vaccine (ZF2001, 25 or 50 μg RBD protein/dose). In Study 1, the main study cohort rats (*n* = 24/group) and the immunogenicity cohort rats (*n* = 12/group) were subjected to caesarean section and full fetal developmental examination on GD 20. In Study 2, the pregnant rats (*n* = 16/group, for the main study and the immunogenicity study) were allowed to deliver naturally, and the growth, development and reproductive performance of offspring rats were monitored from PND 0 to maturity period (postnatal week 11, ~PND77). Blood was collected for measurement of the antibody response in the maternal animals and their offspring as shown above.

An overview of the design of Study 1 (embryo-fetal developmental toxicity, EFD) design is presented in Fig. 4. A total of 144 virgin female Sprague‒Dawley rats (Charles River Laboratories Zhejiang, 8–9 weeks old and 193–231 g at initiation of dosing) were acclimated and randomly assigned to four groups in two cohorts (*n* = 24 per group in the main study cohort, *n* = 12 per group in immunogenicity cohort). The four groups of rats received three doses of vaccine (25 or 50 μg RBD protein/dose), aluminium-based adjuvant or sodium chloride injection intramuscularly in the hindlimb 21 and 7 days prior to mating and gestation day (GD) 6, ~2–3 weeks apart. The animals treated with the 50 μg/dose (50 μg RBD protein/vial), adjuvant and blank control were alternately injected in 1 site of the left or right hindlimb with a volume of 0.5 ml each time, while the volume given to rats treated with 25 μg/dose was halved.

An overview of the design of Study 2 (pre- and postnatal developmental toxicity, PPND) design is also depicted in Fig. 4 (bottom one). Fifty-six virgin female Sprague‒Dawley rats (Charles River Laboratories Zhejiang, 10–12 weeks old and 221–272 g at the initiation of dosing) were acclimated and randomly assigned to two groups (*n* = 28 per group). One group of rats was administered ZF2001 at a dose level of 25 μg RBD protein/dose intramuscularly in the hindlimb 7 days prior to the start of mating, GD 6, GD 20 and postnatal day (PND) 10 (16 pregnant rats), for a total of 4 doses from premating to lactation. At these four time points, another group was administered sodium chloride injection. The animals in each group were alternately injected in 1 site of the left or right hindlimb with 0.5 ml vaccine (25 μg RBD protein/vial) or sodium chloride injection each time. The growth, development and reproductive performance of offspring rats was monitored by various methods described below from PND 0 to postnatal week 11, when the rats of the fertility test subgroup were used to be mated to evaluate the fertility of offspring.

### Observations and measurements

#### Study 1 (EFD)

Body weight, food consumption, and clinical signs were monitored throughout the study. Vaginal smears were collected from the females daily, and pregnancy began when positive evidence of mating (the presence of a copulatory plug) was observed. The day on which positive evidence of mating was found was recorded as gestation day (GD) 0.

Pregnant rats were euthanized on GD 20 via CO_2_ asphyxiation followed by cervical dislocation. In the main study cohort, a gross examination was performed, and the gravid uterus was removed and weighed. Thereafter, the number of corpora lutea, implantation sites, resorptions, and live and dead conceptuses were recorded. Gross evaluation of the placenta was performed, and the placentas were weighed. Live fetuses were removed from the uteri and individually weighed, and the sex was recorded. The body length and tail length of live fetuses were measured individually. Thereafter, live fetuses were euthanized by intraperitoneal injection of sodium pentobarbital (0.1 ml of 5 mg/ml; Hangzhou Dacheng, China) followed by decapitation for blood collection. External evaluation of each fetus was conducted. Approximately 50% of the fetuses were subjected to soft tissue examination, and the other 50% were subjected to skeletal evaluation. During the soft tissue evaluation, fetuses were fixed in Bouin’s solution and subsequently examined by serial sectioning. During the skeletal evaluation, fetuses were peeled, eviscerated, fixed in 95% of ethanol, and stained with Alcian blue and alizarin red staining solution successively. External, soft tissue and skeletal findings were recorded as malformations, variations, or uncategorised abnormalities in general, referring to standardised terminology^34^.

#### Study 2 (PPND)

Throughout the study, the clinical signs, body weight and food consumption of the parental (F0) female rats were recorded. Pregnant females were evaluated for natural delivery parameters, and gross examinations were performed on the anatomy of PND 21. F1 generation pups were reduced to 8/litter with a half male and half female composition on PND4. Their clinical signs, survival, body weight, and physical and reflex development indicators were observed from PND 0 to weaning (PND21). For physical and reflex development indicators, we began to observe auricle separation from PND 1, positive plane from PND 2, negative geotaxis from PND 6, incisor eruption from PND 8, hair emergence from PND 9, auditory shock from PND 11, mid-air correction from PND 11, eye opening from PND 13, pinna unfolding from PND 14, and pupillary reflex starting after eye opening. We recorded the above developmental results for all animals until the whole litter showed positivity. The autonomic activity test (open-field experiment, 5 min) was performed on PND 19 by TopScan^TM^2.0 (Clever Sys., Inc.), and the modified Irwin’s behavioural evaluation (a total of 35 indicators) was performed on PND 20 for 1 pup/sex/litter/group. Six of eight pups per litter were euthanized for gross examination on PND 21. A remaining 1 pup/sex/litter for the fertility test subgroup continued to be fed. The body weight, food consumption, sexual development (the age of vaginal opening and prepuce separation started from PND 27 or PND38, respectively) and fertility of these F1 generation rats were evaluated. During postnatal week 11, one female/litter was cohabited with a nonsibling male within the same treatment group. Upon confirmation of mating, the rats were removed and individually caged. The F1 male rats were necropsied after cohabitation, and the F1 female rats were necropsied on GD 15, and the uterine contents were examined.

### Antibody analysis

During the immunogenicity examination of Study 1, blood samples were collected from F0 females on the day before cohabitation (after receiving 2 vaccinations 21 and 7 days prior to mating) and GD 20 from the jugular vein or abdominal aorta, respectively. In addition, fetal blood samples were collected on GD 20 from arbitrarily selected fetuses by decapitation and samples were subsequently pooled by litter (minimum of 0.5 ml/litter). In Study 2, blood samples were collected from F0 female rats on GD 20 (after 2 vaccinations) and PND 21 (after 4 vaccinations) from the jugular vein or abdominal aorta, respectively. Blood samples were collected from F1 pups on PND 21 and PND 70 from the abdominal aorta or jugular vein, respectively. Samples were collected into tubes without anticoagulant and centrifuged at 3000 rpm for 10 min. The resultant serum was removed and frozen at −80 °C prior to antibody analysis.

For the serological RBD-binding IgG assay in Study 1 and Study 2, ELISA plates were coated overnight with 1 μg/ml RBD protein (Anhui Zhifei Longcom Biopharmaceutical, China) in 1× phosphate buffered solution (PBS), pH 9.6, and blocked in 3% skim milk in PBST. Serum samples were serially diluted and added to each well. Plates were incubated with goat anti-rat IgG-HRP antibody (Abcam, Goat αRat; CAS#: ab97057; 1/2000 dilution) and subsequently developed with 3,3’,5,5’-tetramethylbenzidine (TMB) substrate. Reactions were stopped by the addition of 50 μl of Stopping Solution, and the absorbance was measured at 450 nm using a microplate reader (Molecular Devices, USA). Geometric mean titres (GMTs) were calculated, and the endpoint titres were defined as the highest reciprocal dilution of serum to produce an absorbance greater than the cut-off value (2.1-fold of the background values).

For the pseudovirus neutralising antibody assay in Study 2, serial dilutions of test sera were incubated with SARS-CoV-2 pseudovirus (1/2 dilution) to allow any antigen-specific antibodies to bind to the virus in 96-well tissue culture plates. SARS-CoV-2 pseudovirus (Indian strain delta pseudovirus) was obtained from Beijing Tiantan Biological Products Co., Ltd (Beijing, China). The Huh-7 cells were then transferred into the serum-virus mixture and allowed to be incubated for 20–28 h for infection by the nonneutralized virus. The neutralisation inhibition rate and 50% inhibitory concentration (IC_50_) were calculated.

### Statistics and data analysis

Quantitative data are described as the mean ± SD and were analysed using one-way ANOVA to assess the homogeneity of group variances in Study 1. When the difference among the total groups was statistically significant (*p* < 0.05), the difference between the two groups continued to be compared using the LSD test if the Levene’s test result was not significant or Games-Howell if it was significant. There were only two groups of data in Study 2, and two independent samples *t*-tests were used for significance analysis. Counting data were described as percentages and were analysed using the chi-square (*χ*^2^) test. The relative indicators of fetal growth and development and external, visceral and skeletal morphology were assessed both by litter and fetal individuals. The indicators of fetal body weight, body length and tail length in Study 1 and pup body weight and physical and reflex development signs in Study 2 were assessed by litter to comprehensively evaluate the influence of the litter effect. All statistical analyses were carried out with SPSS 23.0, and results with a *p* value of <0.05 were considered significant.

### Reporting summary

Further information on research design is available in the Nature Research Reporting Summary linked to this article.

## Supplementary information

Supplementary Figures

REPORTING SUMMARY

## Data Availability

All data are also available from the corresponding authors on reasonable request.

## References

[1] World Health Organization. https://www.who.int/.

[2] Gao GF (2021). Science-based COVID-19 vaccine development. Natl Sci. Rev..

[3] Barda N (2021). Effectiveness of a third dose of the BNT162b2 mRNA COVID-19 vaccine for preventing severe outcomes in Israel: an observational study. Lancet.

[4] Halperin SA (2022). Final efficacy analysis, interim safety analysis, and immunogenicity of a single dose of recombinant novel coronavirus vaccine (adenovirus type 5 vector) in adults 18 years and older: an international, multicentre, randomised, double-blinded, placebo-controlled phase 3 trial. Lancet.

[5] Tanriover MD (2021). Efficacy and safety of an inactivated whole-virion SARS-CoV-2 vaccine (CoronaVac): interim results of a double-blind, randomised, placebo-controlled, phase 3 trial in Turkey. Lancet.

[6] Yang S (2021). Safety and immunogenicity of a recombinant tandem-repeat dimeric RBD-based protein subunit vaccine (ZF2001) against COVID-19 in adults: two randomised, double-blind, placebo-controlled, phase 1 and 2 trials. Lancet Infect. Dis..

[7] Sutton D (2021). COVID-19 vaccine acceptance among pregnant, breastfeeding, and nonpregnant reproductive-aged women. Am. J. Obstet. Gynecol. MFM.

[8] Anderson RM, Vegvari C, Truscott J, Collyer BS (2020). Challenges in creating herd immunity to SARS-CoV-2 infection by mass vaccination. Lancet.

[9] Liu H (2020). Why are pregnant women susceptible to COVID-19? An immunological viewpoint. J. Reprod. Immunol..

[10] Jamieson DJ, Rasmussen SA (2022). An update on COVID-19 and pregnancy. Am. J. Obstet. Gynecol..

[11] Jafari M (2021). Clinical characteristics and outcomes of pregnant women with COVID-19 and comparison with control patients: a systematic review and meta-analysis. Rev. Med. Virol..

[12] Zambrano LD (2020). Update: Characteristics of symptomatic women of reproductive age with laboratory-confirmed SARS-CoV-2 infection by pregnancy status - United States, January 22-October 3, 2020. Morb. Mortal. Wkly Rep..

[13] Allotey J (2020). Clinical manifestations, risk factors, and maternal and perinatal outcomes of coronavirus disease 2019 in pregnancy: living systematic review and meta-analysis. BMJ.

[14] Desai P, Kaur G, Dong F, Rodriguez M (2021). COVID-19 vaccine acceptance in pregnancy. Neonatol. Today.

[15] Dai L, Gao GF (2021). Viral targets for vaccines against COVID-19. Nat. Rev. Immunol..

[16] Dai L (2020). A universal design of betacoronavirus vaccines against COVID-19, MERS, and SARS. Cell.

[17] Walls AC (2020). Elicitation of potent neutralizing antibody responses by designed protein nanoparticle vaccines for SARS-CoV-2. Cell.

[18] An Y (2022). A tandem-repeat dimeric RBD protein-based Covid-19 vaccine zf2001 protects mice and nonhuman primates. Emerg. Microbes Infect..

[19] Dai, L. et al. Efficacy and safety of the RBD-dimer-based Covid-19 vaccine ZF2001 in adults. *N. Engl. J. Med.*10.1056/NEJMoa2202261 (2022). 10.1056/NEJMoa2202261 PMC9127771 35507481

[20] World Health Organization. (2005). WHO guidelines on nonclinical evaluation of vaccines. WHO Tech. Rep. Ser..

[21] ICH. ICH Harmonised Guideline S5(R3): detection of reproductive and developmental toxicity for human pharmaceuticals. (2020).

[22] Gruber MF (2003). Maternal immunization: US FDA regulatory considerations. Vaccine.

[23] Bowman CJ (2021). Lack of effects on female fertility and prenatal and postnatal offspring development in rats with BNT162b2, a mRNA-based COVID-19 vaccine. Reprod. Toxicol..

[24] US Food and Drug Administration. Guidance for industry: considerations for developmental toxicity studies for preventive and therapeutic vaccines for infectious disease indications. (2006).

[25] Namdari R (2021). Species selection for nonclinical safety assessment of drug candidates: examples of current industry practice. Regul. Toxicol. Pharmacol..

[26] Klein, S. L., Creisher, P. S. & Burd, I. COVID-19 vaccine testing in pregnant females is necessary. *J. Clin. Invest.* **131**10.1172/JCI147553 (2021). 10.1172/JCI147553 PMC7919709 33444286

[27] Beigi RH (2021). The need for inclusion of pregnant women in COVID-19 vaccine trials. Vaccine.

[28] Burd, I., Kino, T. & Segars, J. The Israeli study of Pfizer BNT162b2 vaccine in pregnancy: considering maternal and neonatal benefits. *J. Clin. Invest.* **131**10.1172/JCI150790 (2021). 10.1172/JCI150790 PMC8245166 34101621

[29] Zaman K (2008). Effectiveness of maternal influenza immunization in mothers and infants. N. Engl J. Med..

[30] Jamieson DJ, Kissin DM, Bridges CB, Rasmussen SA (2012). Benefits of influenza vaccination during pregnancy for pregnant women. Am. J. Obstet. Gynecol..

[31] Trostle ME, Aguero-Rosenfeld ME, Roman AS, Lighter JL (2021). High antibody levels in cord blood from pregnant women vaccinated against COVID-19. Am. J. Obstet. Gynecol. MFM.

[32] De Rose, D. U., Salvatori, G., Dotta, A. & Auriti, C. SARS-CoV-2 vaccines during pregnancy and breastfeeding: a systematic review of maternal and neonatal outcomes. *Viruses* **14**, 10.3390/v14030539 (2022). 10.3390/v14030539 PMC8951373 35336947

[33] Whitehead CL, Walker SP (2020). Consider pregnancy in COVID-19 therapeutic drug and vaccine trials. Lancet.

[34] Makris SL (2009). Terminology of developmental abnormalities in common laboratory mammals (version 2). Congenit. Anom..

