# Supplementary material for: Developmental and reproductive toxicity of a recombinant protein subunit COVID-19 vaccine (ZF2001) in rats
Source: NPJ Vaccines. 2023 May 24;8:74. doi: 10.1038/s41541-023-00673-3 (PMC10206582; doi:10.1038/s41541-023-00673-3)
Supplement: Supplementary file 1 — Supplementary Figures [file 41541_2023_673_MOESM1_ESM.pdf]

# Developmental and reproductive toxicity of a recombinant protein subunit COVID-19 vaccine (ZF2001) in rats

Yisheng Song [1,2,†], Jinjin Shao [1,2,†], Guangbiao She [3,†], Wanqiang Lv [1,2], Guoyu Chen[1,2], Jing Liu [1,2], Lili Zhang [1,2], Chengda Zhang [1,2], Jiahong Wang [1,2], Ruiyu Tian [1,2], Lianpan Dai [4], George F. Gao [4], Enqi Huang [3,*] and Lijiang Zhang [1,2,*]

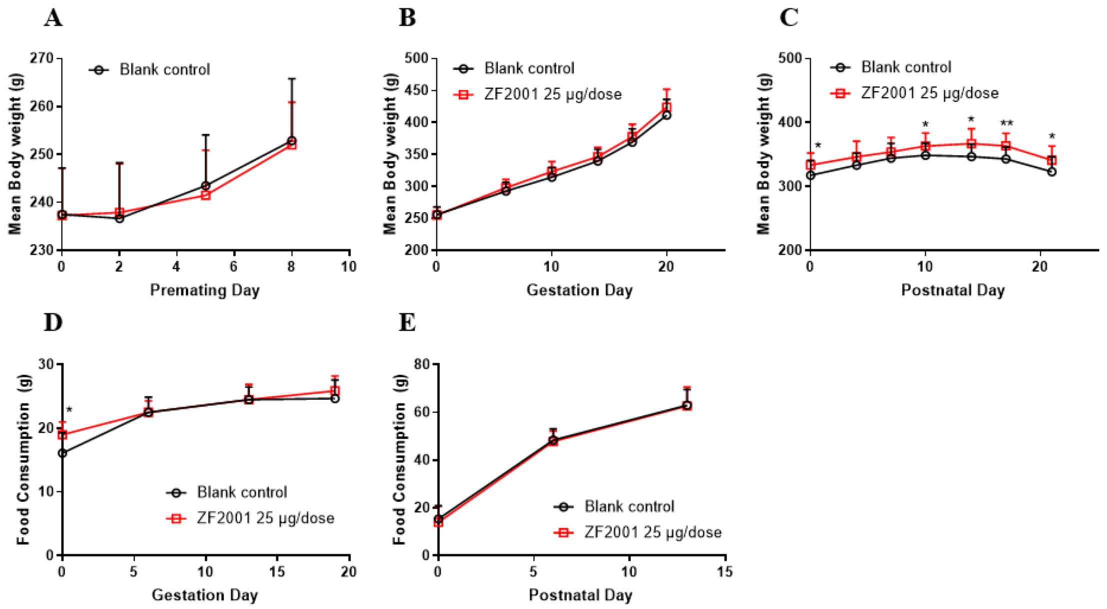


**Supplementary Figure 1 Mean body weight and food consumption of F0 female rats in Study 2.** Data are expressed as the mean ± SD. Compared with blank control group, * indicates one-way ANOVA $P < 0.05$, ** indicates one-way ANOVA $P < 0.01$.

A-C. Body weight of F0 female rats during the premating, gestation, and postnatal days; D-F. Feed consumption of F0 female rats during the gestation, and postnatal days.

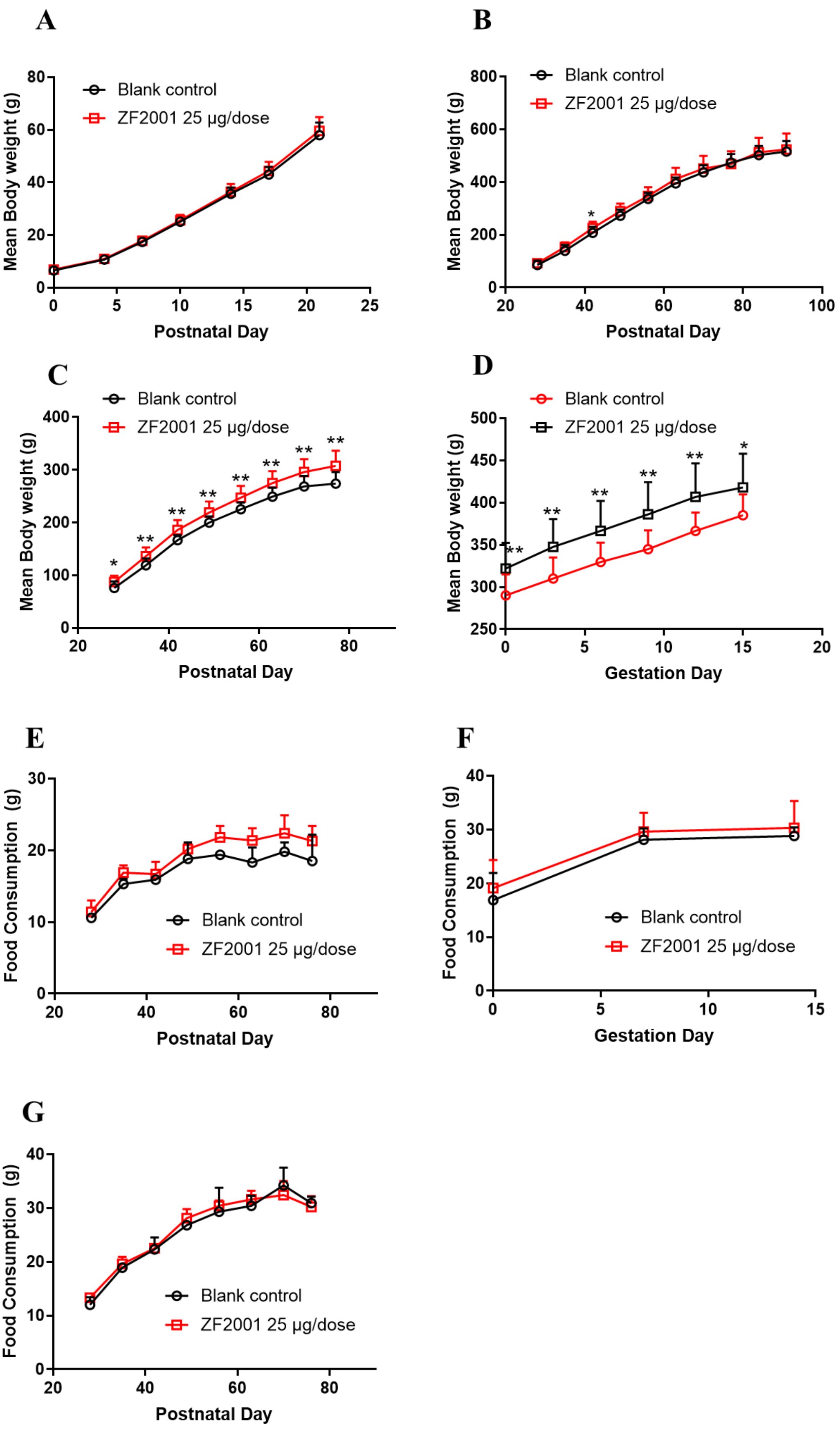
A
Mean Body weight (g)
Postnatal Day
Blank control
ZF2001 25 μg/dose
B
Mean Body weight (g)
Postnatal Day
Blank control
ZF2001 25 μg/dose
C
Mean Body weight (g)
Postnatal Day
Blank control
ZF2001 25 μg/dose
D
Mean Body weight (g)
Gestation Day
Blank control
ZF2001 25 μg/dose
E
Food Consumption (g)
Postnatal Day
Blank control
ZF2001 25 μg/dose
F
Food Consumption (g)
Gestation Day
Blank control
ZF2001 25 μg/dose
G
Food Consumption (g)
Postnatal Day
Blank control
ZF2001 25 μg/dose

**Supplementary Figure 2 Body weight and food consumption of F1 generation rats in Study 2.** Data are expressed as the mean ± SD. Compared with control group, * indicates one-way ANOVA $P < 0.05$, ** indicates one-way ANOVA $P < 0.01$. A. Body weight of F1 generation rats during postnatal days. B. Body weight of F1 male rats after weaning. C. Body weight of F1 female rats after weaning. D. Body weight of F1 female rats during gestation days. E. Food consumption of F1 female rats after weaning. F. Food consumption of F1 female rats during gestation day. G. Food consumption of F1 male rats after weaning.
