# Supplementary material for: Developmental and reproductive toxicity of a recombinant protein subunit COVID-19 vaccine (ZF2001) in rats
Source: NPJ Vaccines. 2023 May 24;8:74. doi: 10.1038/s41541-023-00673-3 (PMC10206582; doi:10.1038/s41541-023-00673-3)
Supplement: Supplementary file 2 — REPORTING SUMMARY [file 41541_2023_673_MOESM2_ESM.pdf]

Corresponding author(s): Lijiang Zhang
Last updated by author(s): May 5, 2023

Reporting Summary

Nature Portfolio wishes to improve the reproducibility of the work that we publish. This form provides structure for consistency and transparency in reporting. For further information on Nature Portfolio policies, see our Editorial Policies and the Editorial Policy Checklist.

Statistics

For all statistical analyses, confirm that the following items are present in the figure legend, table legend, main text, or Methods section.

n/a	Confirmed	
☐	☒	The exact sample size (*n*) for each experimental group/condition, given as a discrete number and unit of measurement
☐	☒	A statement on whether measurements were taken from distinct samples or whether the same sample was measured repeatedly
☐	☒	The statistical test(s) used AND whether they are one- or two-sided<br>*Only common tests should be described solely by name; describe more complex techniques in the Methods section.*
☒	☐	A description of all covariates tested
☐	☒	A description of any assumptions or corrections, such as tests of normality and adjustment for multiple comparisons
☐	☒	A full description of the statistical parameters including central tendency (e.g. means) or other basic estimates (e.g. regression coefficient) AND variation (e.g. standard deviation) or associated estimates of uncertainty (e.g. confidence intervals)
☐	☒	For null hypothesis testing, the test statistic (e.g. *F*, *t*, *r*) with confidence intervals, effect sizes, degrees of freedom and *P* value noted<br>*Give P values as exact values whenever suitable.*
☒	☐	For Bayesian analysis, information on the choice of priors and Markov chain Monte Carlo settings
☒	☐	For hierarchical and complex designs, identification of the appropriate level for tests and full reporting of outcomes
☒	☐	Estimates of effect sizes (e.g. Cohen's *d*, Pearson's *r*), indicating how they were calculated

Our web collection on statistics for biologists contains articles on many of the points above.

Software and code

Policy information about availability of computer code

Data collection	All data were recorded with SPSS 23.0
Data analysis	All statistical analyses were carried out with SPSS 23.0

For manuscripts utilizing custom algorithms or software that are central to the research but not yet described in published literature, software must be made available to editors and reviewers. We strongly encourage code deposition in a community repository (e.g. GitHub). See the Nature Portfolio guidelines for submitting code & software for further information.

Data

Policy information about availability of data

All manuscripts must include a data availability statement. This statement should provide the following information, where applicable:

- Accession codes, unique identifiers, or web links for publicly available datasets
- A description of any restrictions on data availability
- For clinical datasets or third party data, please ensure that the statement adheres to our policy

All data are also available from the corresponding authors on reasonable request.

Research involving human participants, their data, or biological material

Policy information about studies with human participants or human data. See also policy information about sex, gender (identity/presentation), and sexual orientation and race, ethnicity and racism.

Reporting on sex and gender	n/a
Reporting on race, ethnicity, or other socially relevant groupings	n/a
Population characteristics	n/a
Recruitment	n/a
Ethics oversight	n/a

Note that full information on the approval of the study protocol must also be provided in the manuscript.

Field-specific reporting

Please select the one below that is the best fit for your research. If you are not sure, read the appropriate sections before making your selection.

☒ Life sciences ☐ Behavioural & social sciences ☐ Ecological, evolutionary & environmental sciences

For a reference copy of the document with all sections, see nature.com/documents/nr-reporting-summary-flat.pdf

Life sciences study design

All studies must disclose on these points even when the disclosure is negative.

Sample size	Study1: A total of 144 virgin female Sprague–Dawley rats (Charles River Laboratories Zhejiang, 8-9 weeks old and 193-231 g at initiation of dosing) were acclimated and randomly assigned to four groups in two cohorts (n = 24 per group in the main study cohort, n = 12 per group in immunogenicity cohort).<br>Study 2: Fifty-six virgin female Sprague–Dawley rats (Charles River Laboratories Zhejiang, 10-12 weeks old and 221-272 g at the initiation of dosing) were acclimated and randomly assigned to two groups (n = 28 per group).
Data exclusions	n/a
Replication	n/a
Randomization	randomly assigned
Blinding	n/a

Reporting for specific materials, systems and methods

We require information from authors about some types of materials, experimental systems and methods used in many studies. Here, indicate whether each material, system or method listed is relevant to your study. If you are not sure if a list item applies to your research, read the appropriate section before selecting a response.

Materials & experimental systems

n/a	Involved in the study	
☐	☒	Antibodies
☒	☐	Eukaryotic cell lines
☒	☐	Palaeontology and archaeology
☐	☒	Animals and other organisms
☒	☐	Clinical data
☒	☐	Dual use research of concern
☒	☐	Plants

Methods

n/a	Involved in the study	
☒	☐	ChIP-seq
☒	☐	Flow cytometry
☒	☐	MRI-based neuroimaging

Antibodies

Antibodies used	goat anti-rat IgG-HRP antibody (Abcam, Goat αRat; CAS#: ab97057; 1/2000 dilution)
Validation	Two times for the test

Animals and other research organisms

Policy information about studies involving animals; ARRIVE guidelines recommended for reporting animal research, and Sex and Gender in Research

Laboratory animals	Sprague–Dawley rats
Wild animals	n/a
Reporting on sex	Female
Field-collected samples	Rats were provided with a complete rodent breeding diet and locally sourced water (softened and filtered) ad libitum. Environmental conditions throughout the studies were set to maintain a relative humidity of 43% to 56% and temperature of 21.5? to 23.0? along with the room lighting set to provide a 12 h light/dark cycle.
Ethics oversight	The facility where these studies were conducted is accredited by the Association for Assessment and Accreditation of Laboratory Animal Care International (AAALAC, #001489). All animal care and experimental procedures were conducted in compliance with guidelines for the care and use of laboratory animals and the relevant regulations of the Institutional Animal Care and Use Committee (IACUC) and approved by the IACUC (approval number: GLP-2020-105/1 and GLP-2021-076).

Note that full information on the approval of the study protocol must also be provided in the manuscript.
